# Surface analysis of Metrohm BT220 screen-printed electrodes through electrochemical techniques: importance of pretreatment

**DOI:** 10.3389/fchem.2025.1602365

**Published:** 2025-09-17

**Authors:** Chun Keat Khor, Sherab Denker, Anna Ignaszak

**Affiliations:** Department of Chemistry, University of New Brunswick, Fredericton, NB, Canada

**Keywords:** gold screen printed electrode, electropolishing, electrochemical capacitance spectroscopy, electrode stability, biosensor

## Abstract

The pandemic that happened a few years ago has made many people aware of the importance of early detection for diseases. Hence, interest in research topics related to biosensors development, especially for point-of-care devices, is as high as it can be. To develop an electrochemical biosensor that meets technical requirements such as miniaturization and compactness in a single piece, many researchers have transitioned from a classical three-electrode system with the typical glass electrochemical cell equipped with large and separated electrodes to screen-printed electrodes (SPEs) and their corresponding accessories, allowing for small sample volume. Gold SPEs can be easily fabricated in large quantities and modified with various biological molecules through the formation of self-assembled monolayers, providing extremely sensitive responses to electrochemical signals and making them an attractive candidate for biosensor designs. In this work, an important pretreatment step, electropolishing in sulfuric acid, was investigated for gold SPE supplied by Metrohm, product BT220. Electrochemical capacitance spectroscopy (ECS) was employed to determine capacitance, which was correlated with changes in surface area, thereby providing insight into how various parameters of cyclic voltammetry (CV) used in electropolishing influence the reproducibility of the pre-treatment process. To optimize the electropolishing process of gold SPE, we have found that (a) the number of CV cycles during electropolishing should be set to ensure that all electrodes reached the same gold reduction peak current, which provides both the very low RSD for electrochemical quantitate of a baseline electrode (i.e., capacitance and active surface area below 2.9% and 1.9%, respectively) (b) the reference electrode incorporated in SPE is not stable in ferricyanide/ferrocyanide solutions, which are frequently used as a standard redox probe in electrochemical biosensors; and (c) this type of SPE should not be used in solutions containing ethanol, the solvent commonly used to dissolve thiolate blocking agents. This analysis provides insight into how to optimize the SPE’s pre-treatment, ensuring the sensor platform is consistent and the surface is reproducible before biological modifications, which in turn yields more steadfast results for biosensor development.

## 1 Introduction

Screen printed electrodes have garnered a lot of attention throughout recent decades. Searching for the keyword “screen printed electrodes” in peer-reviewed literature databases (such as Scopus or Scifinder) can retrieve as many as 2,500 articles and review papers published between 2019 and 2024. One reason behind the rise in popularity of SPE is the ease of fabrication in large quantities compared to classical disk electrodes. Zamani et al. (2023) provided a detailed explanation and comparison of various fabrication processes for gold SPE. In brief, ink paste, such as carbon paste or gold paste, is applied to a substrate, usually polymers or ceramics, and then cured at various temperatures to produce a variety of SPEs for different applications. Compared to classical disk electrodes, where a rod of carbon or gold is usually encased in epoxy to produce a single electrode, mass-producing SPE is easier. Additionally, external counter and reference electrodes are purchased separately for the disk electrode setup, whereas the counter and reference electrodes are printed alongside the working electrode surface on a single substrate, thereby simplifying the electrochemical setup.

Among all the different types of screen printed electrodes (SPE), gold SPEs are typically preferred for the development of biosensors. This is primarily attributed to the ease with which a self-assembled monolayer (SAM) of a thiolated compound forms on a gold surface. SAM is the spontaneous adsorption of molecular monolayers on a solid substrate (Redondo-Gómez et al., 2024). The phenomenon of disulfide adsorption on gold surfaces was first discovered in the 1980s (Grönbeck et al., 2000), and numerous studies have been conducted since then to expand our understanding of this phenomenon (Alves et al., 1992; Bürgi, 2015; Cossaro et al., 2008; Grönbeck et al., 2000). Taking advantage of this simple property of thiol functional groups, various research groups have developed multiple strategies to functionalize different biological molecules, such as antibodies (Fischer et al., 2009; Nowinski et al., 2012), DNA (Barou et al., 2012; Enomoto et al., 2018; Liu et al., 2010; Monteiro and Koper, 2019), and peptides (Brinãs et al., 2012; Nowinski et al., 2012), on gold electrode surfaces. Coupled with the fact that various companies, such as Sigma Oligo, Integrated DNA Technologies, and Thermo Fischer Scientific, provide services that synthesize thiolated biological molecules, electrochemical biosensor research has become much more accessible.

One crucial step before running an electrochemical experiment on gold surfaces is the pretreatment process. This is to enhance the surface quality by removing any contamination that may impact the results during electrochemical studies (Fischer et al., 2009). For classical gold disk electrodes, some common pretreatment processes include, but not limited to, mechanical polishing on a polishing pad with alumina slurries (Fischer et al., 2009; Monteiro and Koper, 2019), dipping the gold surface in a mixture of concentrated H_2_SO_4_ and H_2_O_2_ solution (piranha solution. Danger! Strong oxidizer) (Fischer et al., 2009; Liu et al., 2010), electrochemical pretreatment through cyclic voltammetry (CV) in low concentration sulfuric acid (Barou et al., 2012; Fischer et al., 2009), and exposure to ultraviolet light (Enomoto et al., 2018; Fischer et al., 2009). Despite the popularity of gold SPE, the pretreatment process still varies from paper to paper, even when researchers imply the same type of gold electrodes. Taking Metrohm BT220 gold SPE as an example, the go-to pretreatment process is a simple electrochemical activation of the gold surface using H_2_SO_4_ solution, as recommended by the SPE’s manufacturer. However, the parameters used during the CV process still have minor variations between different research groups. In Table 1, we have listed several Metrohm BT220 electropolishing protocols reported in literature to provide an overview of various approaches towards this critical step in biosensor prototyping.

**TABLE 1:** Parameters used during electrochemical pretreatment via cyclic voltammetry (CV) for a gold Metrohm BT220 screen printed electrode in H_2_SO_4_.

Year	H_2_SO_4_ Conc. (M)	No. of CV cycles	Potential range (V)	Scan rate (V/s)
2014 (Martín-Fernández et al., 2014)^a^	0.5	Until stable	0.00 to 1.25	0.1
2015 (Fátima Barroso et al., 2015)	0.1	Until stable	0.00 to 1.60	0.1
2015 (Yu et al., 2015)	0.1	N/A	0.00 to 1.25	0.1
2018 (García-Mendiola et al., 2018)	0.1	10	−0.20 to 1.20	0.1
2018 (Manzano et al., 2018)^b^	0.5	10	−0.10 to 1.20	0.1
2019 (Díaz-Fernández et al., 2019)^a^	0.5	10	0.00 to 1.30	0.1
2019 (Guerrero et al., 2019)	0.1	10	0.00 to 1.25	0.1
2021 (Sánchez-Salcedo et al., 2021)^a^	0.5	About 10	0.00 to 1.30	0.1
2022 (Zamani et al., 2022)	0.5	10	−0.10 to 1.30	0.1
2022 (Zargartalebi et al., 2022)	0.5	30	0.00 to 1.10	0.1
2023 (Jamal et al., 2023)	0.5	22	−0.20 to 1.20	0.1
2024 (Asaei et al., 2024)^a^	0.5	6	−0.20 to 1.30	0.1
2024 (Kim et al., 2024)	0.5	N/A	0.00 to 1.20	N/A
2024 (Kavetskyy et al., 2024)^c^	0.5	30	0.00 to 1.70	0.1
2024 (Martínez-Periñ et al., 2024)	0.5	10	−0.20 to 1.20	0.1

^a^ Electrodes were cleaned with EtOH, and water, and dried under nitrogen gas BEFORE, performing CV.

^b^ Electrodes were rinsed with EtOH, and water AFTER, performing CV.

^c^ The Ag reference electrode and the counter electrode were disconnected, and external reference and counter electrodes were used instead.

In this study, we investigated how changes in experimental settings used for the electrochemical polishing (pretreatment) of the gold working electrode can directly affect the gold surface and thus influence the results of electrochemical testing. A deeper look and evaluation of the effect of electropolishing conditions should enable other users of similar electrodes to tune the surface confidently, prepare the sensor platform for their experiments, and reduce inconsistencies in experimental results.

## 2 Materials and methods

Potassium hexacyanoferrate (II) trihydrate (99%), potassium hexacyanoferrate (III, 98%), magnesium chloride hexahydrate (99%), and phosphate buffer saline (PBS, 10X) were purchased from Sigma Aldrich. Sulfuric acid (98%) was purchased from Thermo Fischer Scientific. Ethanol (95% vol) was purchased from Commercial Alcohols. These chemicals were used without further purification. Screen-printed gold electrodes (C220BT) were purchased from Metrohm DropSens.

SEM images were taken at the UNB Microscopy and Microanalysis Facility with a JEOL JSM-6400 Scanning Electron Microscope. The microscope has an EDAX Genesis 4,000 Energy Dispersive X-ray (EDS) analyzer. EDS analysis was performed at an accelerating voltage of 15 kV and a beam current of 1.5 nA, with a working distance of 14 mm and a collection time of 50 s per analysis point.

All electrochemical experiments were carried out using a PalmSens4 potentiostat. The SPEs were connected to the potentiostat via Metrohm Dropsens cable connector for SPEs (catalog code: CAC). The experiments were controlled using the PSTrace software (version 5.9.4515). After removing the SPEs from the box, they were first examined for any visible scratches on their surface. Before the experiments, the electrodes were rinsed with 4 mL of DI water to remove dust, followed by blow-drying with a gentle stream of N_2_ gas for 10 s. For sulfuric acid cleaning, 100 μL of 0.5 M was cast to cover all three electrode surfaces and waited 5 min. Then, cyclic voltammograms were recorded from 0.0 V to 1.1/1.2/1.3 V at 0.1/0.2/0.3 V/s for different numbers of cycles. After acquiring the CVs, the 100 μL drop of H_2_SO_4_ was removed by suction with a piece of Kimwipe without touching the electrode surface. Afterward, the electrodes were rinsed with 4 mL of DI water and blow-dried with N_2_ gas. The cleaning process was always carried out before any experiment to prevent any possible changes to the gold surface after leaving it in the air for too long.

The surface area of the working electrodes was calculated following the method proposed by Trasatti and Petrii (1992). Briefly, the obtained CVs were converted into current vs. time graphs using the PSTrace software. Then, the area under the Au reduction peak was integrated using the software’s built-in ‘integration’ function. The product of this peak integration is a charge Q, which was then divided by 3.84 C/m^2^ to obtain the surface area in cm^2^. A detailed explanation of this method is also outlined on the PalmSense website (PalmSens Compact Electrochemical Interfaces, 2021).

To acquire electrochemical impedance spectra (EIS), 100 μL of PBS (1X) with 5 mM of MgCl_2_ solution was drop cast on the SPE surface. The EIS measurements were carried out at 0.0 V d.c (direct current) potential with the a.c. (alternating current) potential amplitude of 10 mV and in the frequency range from 100 kHz to 1.0 Hz. EIS data were then fitted to the proposed electrical equivalent circuit using the ‘circuit fitting’ function available in the PSTrace software. The equivalent circuit representing the electrode-electrolyte interface comprises a resistor connected in series to a constant phase element (see Supplementary Figure S1). Equation 1 was used to calculate the capacitance, as outlined by Chang (2022):

$$Capacitance=\frac{{\left(R_{s}Y_{0}\right)}^{\frac{1}{n}}}{R_{s}}$$ (1)

Where *R*_*s*_ is the solution resistance, *Y*_*0*_ and *n* are parameters characterizing the constant phase element. Both *R*_*s*_ and *Y*_*0*_ are denoted as R and Q, respectively, in the PSTrace software.

Further analysis and calculations were performed using Microsoft Excel (see raw data). The standard deviation was estimated using the ‘= STDEV.S’ function, and the error percentage was calculated by dividing the average by the standard deviation for each set of data. All graphical images in this article were generated from Microsoft Excel and Origin 2018 software.

## 3 Results

**FIGURE 1:**
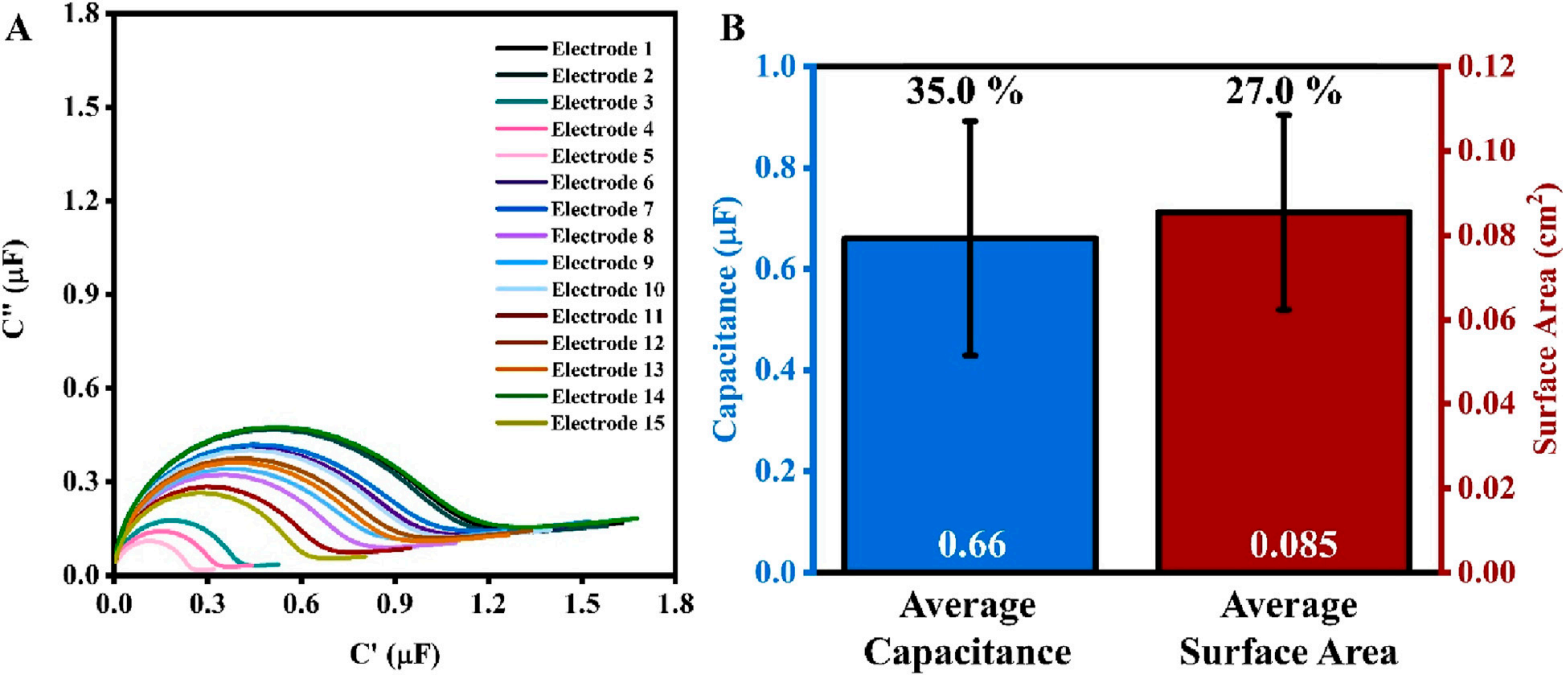
**(A)** Electrochemical capacitance spectra (ECS) for 15 electrodes of the same box. **(B)** Bar chart for the average values of capacitance (blue, left) and surface area (red, right) of the 15 electrodes. The values in white at the base of the bar charts are the average values of capacitance and surface area, while the percentage values (in black) above are the percent error (n = 15).

To better understand the surface of the working electrodes before any pretreatment, 15 SPEs were first rinsed with 4 mL of DI water, dried with N_2_ gas, and subjected to impedance analysis in PBS (1X) and 5 mM MgCl_2_ mixture (Figure 1A). The calculated value of capacitance for these 15 SPEs ranges between 0.22 μF and 0.93 μF, with an average of 0.66 (±35%) μF. Following the impedance analysis, two cycles of cyclic voltammogram were recorded in 0.5 M H_2_SO_4,_ and the surface area was calculated as outlined in the section ‘Materials and Methods’ (Figure 1B). The surface area of electrodes ranges between 0.04 cm^2^ to 0.11 cm^2^, averaging at 0.09 (±27%) cm^2^. Note that the results presented in Figure 1 refer to bare SPEs that were not electropolished before the CV and EIS tests. This was to demonstrate the viability and importance of pretreatment, as discussed in the following sections.

The electrolyte used for EIS analysis that is PBS (1X) with 5 mM of MgCl_2_ does not contain redox active species, and the applied voltage (V_d.c_ = 0.0 V, V_a.c._ = 0.01 V) is outside of the gold electrochemical activity, therefore no redox processes are expected to take place in this experiment.

The electrolyte ions form an electrical double layer near the working electrode surface (see Supplementary Figure S5 for a simple schematic representation). This ion arrangement can be compared to a parallel plate capacitor, in which the capacitance is directly proportional to the area as per Equation 2:

$$Capacitance=\frac{\varepsilon \varepsilon_{0}A}{d}$$ (2)

**FIGURE 2:**
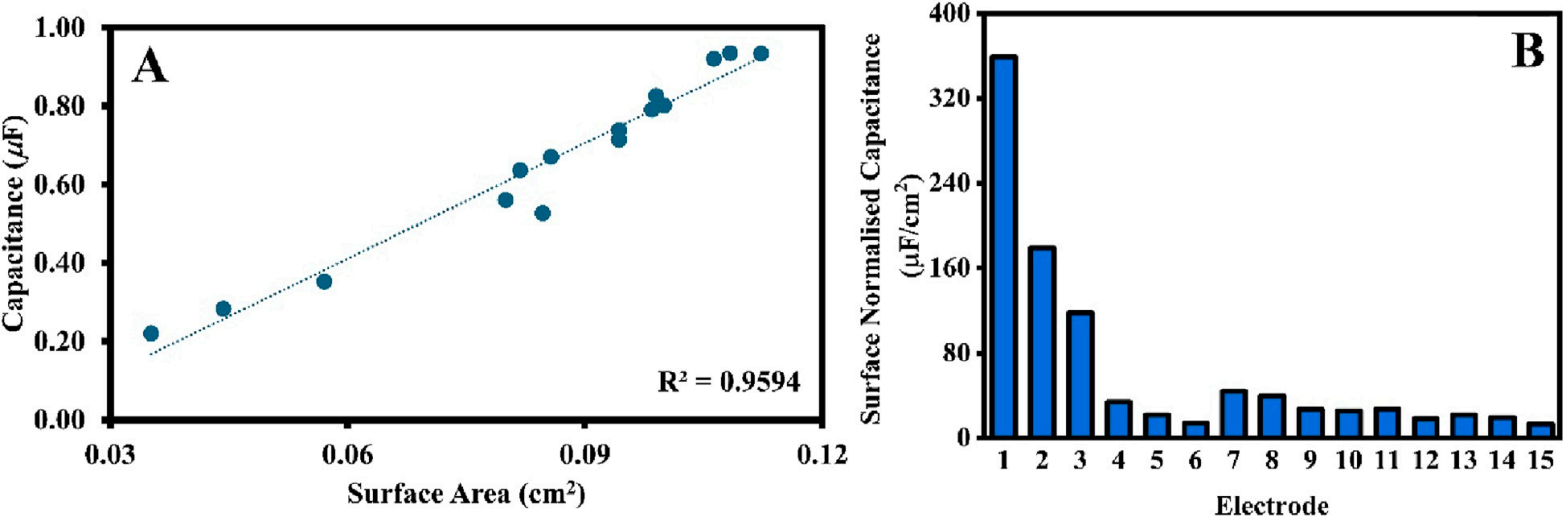
**(A)** A scatter plot of capacitance (calculated from ECS) against surface area (calculated from CV) for 15 SPEs from the same box. The R^2^ value were generated by Microsoft Excel. **(B)** The surface normalized capacitance of the same 15 SPEs.

Where *ε* is the relative permittivity of dielectric, *ε*_*0*_ is the relative permittivity of vacuum, A is the area of the parallel plates, and d is the distance between the two parallel plates. The correlation defined in Equation 2 was validated and projected in Figure 2A. The average capacitance of 15 electrodes, calculated from ECS data (Figure 1A), was plotted against the working electrode surface area, quantified using cyclic voltammetry. The linear dependence, with a high variance of R^2^ = 0.96, demonstrates that electrochemical quantities are well-correlated and follow a linear relationship as defined in Equation 2, which also validates the use of these two electrochemical methods (ECS and CV) to quantify the surface area of the working electrode. The results presented in Figures 1, 2 serve as baselines for comparison with the analysis of the influence of the conditions applied in electropolishing on the quality and reproducibility of the working electrodes.

### 3.1 An effect of the cyclic voltammetry conditions on the gold surface evaluation

#### 3.1.1 An effect of the number of CV cycles

**FIGURE 3:**
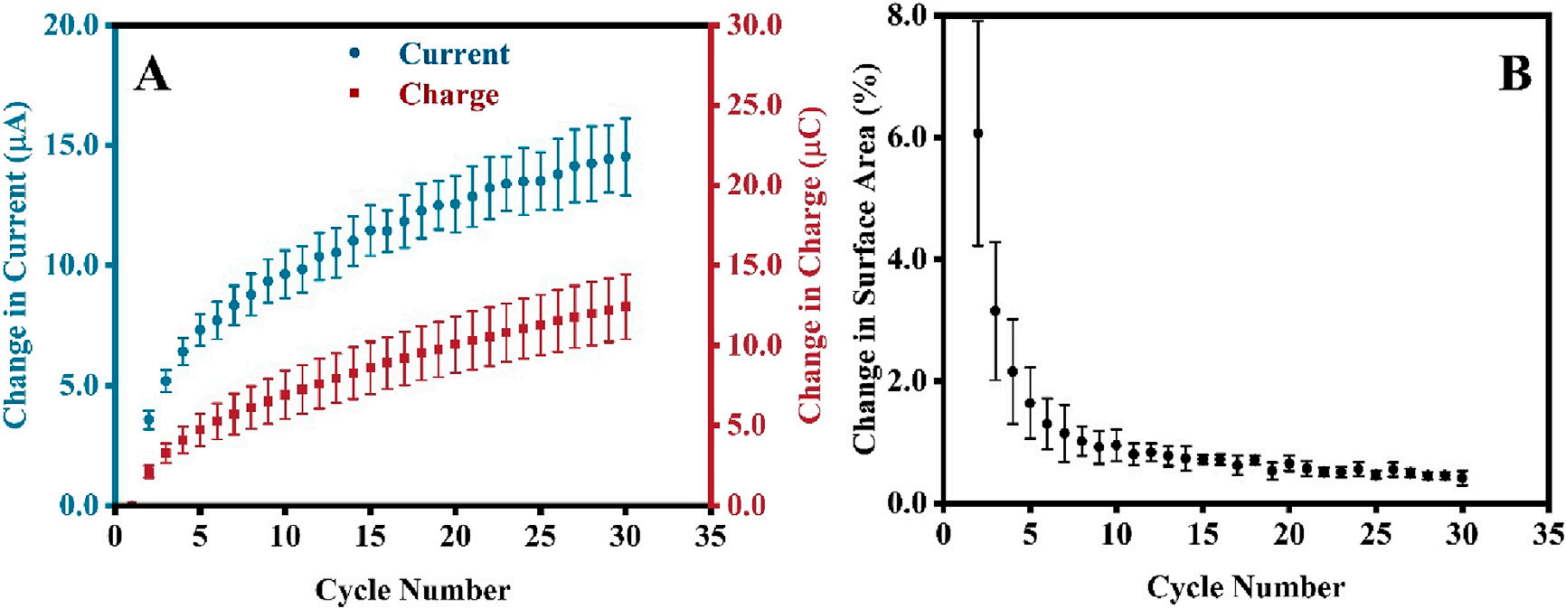
**(A)** Changes in the gold reduction peak current, *δI*, and chargesurface area, *δSA* **(B)** as a function of the number of CV scans. Note that for this experiment, new SPEs were analyzed (not the one from the preceding section), and each experiment was repeated 5 times; therefore, error bars are based on 5 repetitions.

Since CV is used to alter the surface of the electrode, understanding how different parameters of the pretreatment process will affect the surface area is equally important. Firstly, the number of CV scans in the electropolishing was correlated with the electrochemically active surface of the electrodes. An increase in the absolute change in the gold reduction peak current, *δI*, was observed with an increasing number of cycles (Figure 3A, blue circle). The change in current was calculated using Equation 3:

$$\delta I=I_{n}-I_{1}$$ (3)

Where *I*_*n*_ is the peak current and charge at cycle *n* while *I*_*1*_ is the peak current and charge at cycle one. To further understand how these changes affect the surface area, these CVs were again converted to current against the time plot, and the gold reduction peaks were integrated to extrapolate the changes in charges, *δQ* (see Figure 3B, red square), which is calculated using the similar formulae as shown in Equation 4:

$$\delta Q=Q_{n}-Q_{1}$$ (4)

Figure 3B shows that the percent change in surface area decreases with the increasing number of CV scans. The percentage change in surface area, *δSA*, was calculated using Equation 5:

$$\delta SA=\frac{SA_{n}-SA_{n-1}}{SA_{n-1}}\times 100\;\%$$ (5)

Where *SA*_*n*_ and *SA*_*n-1*_ are the surface area values of cycle *n* and *n-1*, respectively. Notably, the initial surface of the electrodes was slightly different; however, the percentage change during the first few cycles (cycles 1–10) varied significantly when compared between all SPEs, leading to the large RSD, as indicated by the magnitude of the error bars in Figure 3B. However, as the electrodes were subjected to more CV cycles, the percentage changes became stagnant; hence, the values became more consistent, resulting in a minor RSD (after the 10th cycle). Since only the gold particles at the surface are affected by the treatment, in electropolishing, the dissolution of the surface gold and the formation of gold oxide layer eliminate the morphological differences of the outer gold deposit introduced during manufacturing and storage. With the increasing number of CV scans, the morphology of most outer deposits is very similar, resulting in low RSD and a reproducible δSA after 10 scans (Figure 3B). We have analyzed the surface of bare and CV-electropolished gold surface (Supplementary Figure S2B) using high-resolution SEM but cannot distinguish any changes in surface morphology to support the above statement adequately. Any further magnification and analysis at the nanoscale level of individual particles might not encapsulate the overall morphology of all gold particle surfaces. Furthermore, the single-particle surface might be too smooth to see differences at that scale.

Unlike the error bars in Figure 3B, the RSD calculated for five electrodes in each experiment increased (error bars in Figure 3A) when the direct readings of the Au reduction peak current were taken into account. This is because the change in peak current does not account for peak broadening, which is observed in the overlay of thirty CVs and current against time graphs in Supplementary Figures S3, S4, respectively. The product of peak integration in Figure 3B recognizes the peak broadening, resulting in significantly reduced RSD. This must be taken into consideration for the validation of the electropolishing process.

#### 3.1.2 An effect of the scan rate and the positive potential limit in CV electropolishing

**FIGURE 4:**
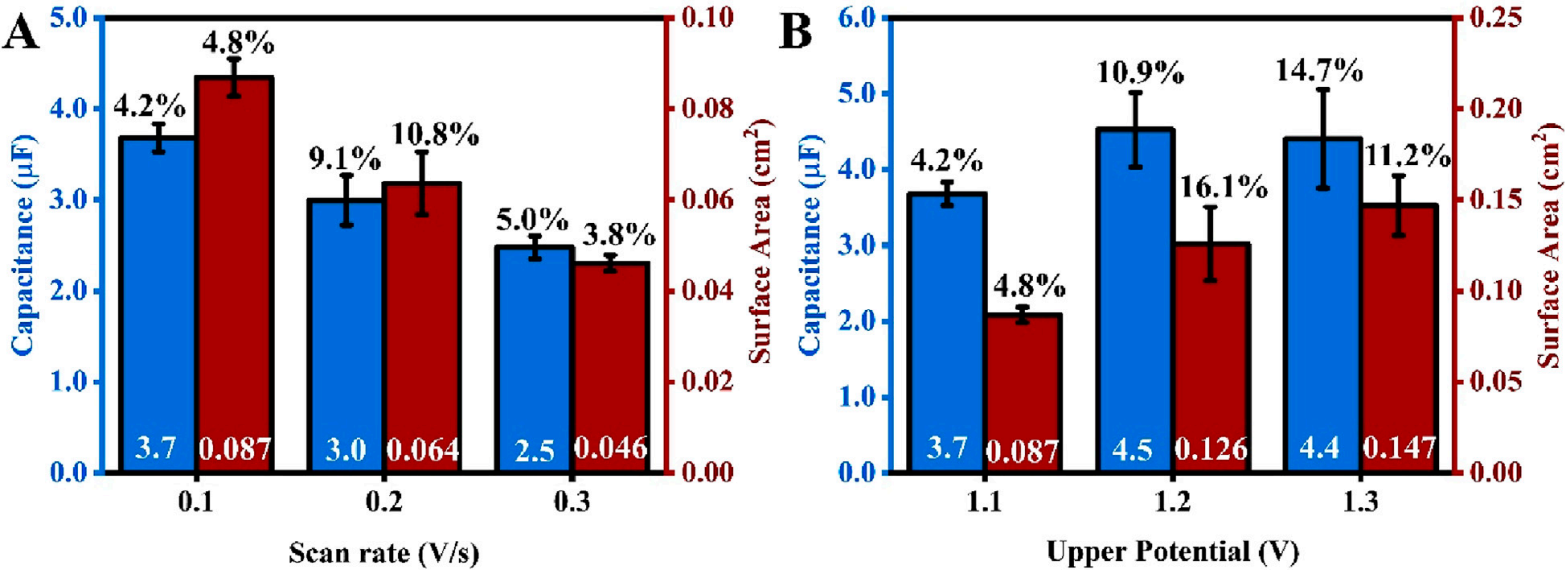
An average capacitance (blue) and surface area (red) of SPEs electropolished at different potential scan rates **(A)** and different positive potential limits **(B)**. The values in white at the base of the bar charts are the average values of capacitance and surface area, while the percentage values (in black) above are the %RSD from five repetitions (number of electrodes for each test, n = 5). The scan rate for experiments projected in Figure 3B is 0.1 V/s.

Figure 4A illustrates that both the surface area and capacitance decrease as the scan rate increases. This observation aligns with the findings of Chevreko et al., who conducted extremely meticulous work to elucidate the mechanism behind gold dissolution and oxide formation in H_2_SO_4_ (Cherevko et al., 2013). In this analysis, researchers noted that both gold dissolution and oxide formation occur simultaneously. An oxide layer forms first, which passivates the gold surface. Concurrently, at a very similar potential, a place-exchange between gold ions and gold oxide occurs, which disturbs the passivated gold layer, exposing the gold surface to the electrolyte and facilitating gold dissolution. The combination of these competing processes contributes to the roughening of the gold surface. However, at a higher scan rate, the time during which gold particles are prone to dissolution and oxide formation is shorter, resulting in a smaller surface area. As shown in Equation 1, since capacitance is directly proportional to surface area, a smaller capacitance value was also observed at the higher scan rate (Figure 4A).

While analyzing the trend in Figure 4A, one can recognize that for the type of screen printed electrodes investigated in this work, the decrease in both surface area and capacitance can be explained with the support of observations reported by Chevreko and colleagues. Given that the gold electropolishing process is sensitive to the composition of the etching medium and the applied potential regime, it is essential to consider that the reactivity and dissolution of gold are strongly influenced by its initial surface roughness and the level of initial surface contamination. This varies significantly between flat gold disks, rods, and foils, as compared to porous gold prints.

Figure 4B shows that both the surface area and capacitance increase as the upper potential limit of the CV increases. This trend also aligns with Chevreko et al., who observed a higher amount of gold dissolution as the upper possible limit increased, resulting in a rougher surface and an increase in surface area. Although the capacitance increased from 1.1 V to 1.2 V, it remained constant from 1.2 V to 1.3 V. This does not align well with the understanding gained from Equation 1. One plausible explanation for this observation is that the solution capacitance is influenced by the concentration of ions in the electrolyte solution (Tajparast and Glavinović, 2018). During the ECS experiment, the same concentration of salt solution was used when testing 1.2 V and 1.3 V. Despite having an increase in surface area after treating the gold surface with higher upper potential (1.3 V), most of the ions could be aligned on the surface for both 1.2 V and 1.3 V, leading to similar capacitance values. Also, at higher anodic potentials, the characteristics of the gold oxide layer or the double-layer structure might alter, leading to a non-linear relationship between capacitance and ECSA. Significant surface roughening or damage at 1.3 V could also create complexities that a basic parallel-plate capacitor model does not account for. Additionally, the percentage relative standard deviation (RSD) increases when a higher potential limit is applied. This is related to the increase in current magnitude for the gold reduction peak between each subsequent cyclic voltammetry (CV) cycle, thereby making it harder to obtain a consistent surface across five electrodes.

**FIGURE 5:**
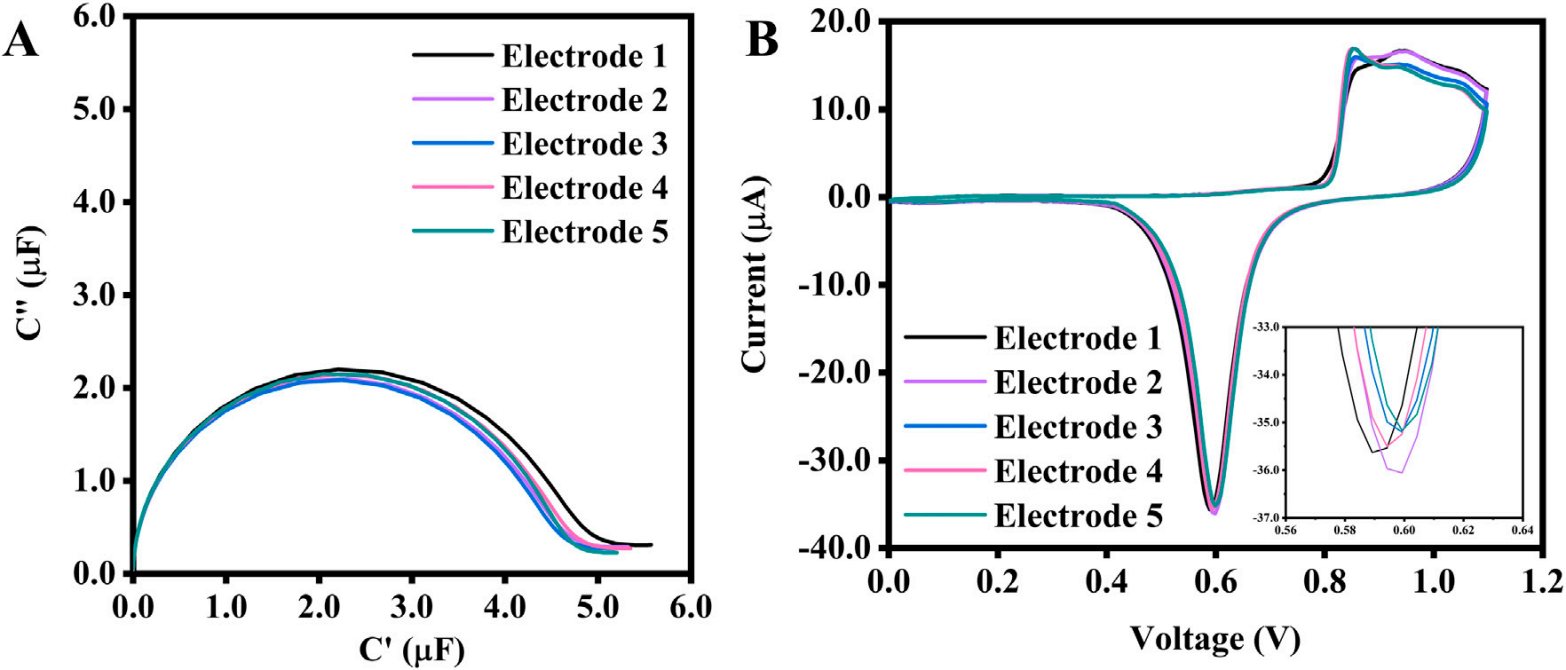
**(A)** ECS of 5 SPEs electropolished with the same scan rate and potential range, but with a different number of CV scans to achieve the same Au reduction peak current of −35.0 μA, and their corresponding CVs **(B)**. The inset is a zoomed-in image of the reduction peak. The potential range for the CV is between 0.0 V and 1.1 V at a scan rate of 0.1 V/s. The number of cycles needed to reach −35.0 μA for electrodes No. 1 to 5 are 5, 5, 6, 8, and 10, respectively.

Based on the observations, the surface area of the working electrode can be controlled to some extent during the pretreatment process, provided that the reduction current values remain constant. To demonstrate this, five SPEs underwent a pretreatment process with the same parameters (an upper potential of 1.1 V at a rate of 0.1 V/s). The number of CV cycles was varied to ensure that all electrodes reached the same reduction peak current (Figures 5A, B), and electropolishing was stopped once all electrodes reached −35.0 µA (see Figure 5B). The calculated surface area averages were 0.095 cm^2^, with an error percentage as low as 2.9%. The average capacitance obtained from ECS was 4.26 μF, with a 1.9% error across five electrodes. This test concluded that to achieve the most consistent capacitance, as confirmed by its very low %RSD, the recommended electropolishing should use the Au peak reduction current as a reference point to consistently pretreat the SPE.

### 3.2 Stability of electrode

#### 3.2.1 Reference electrode

**FIGURE 6:**
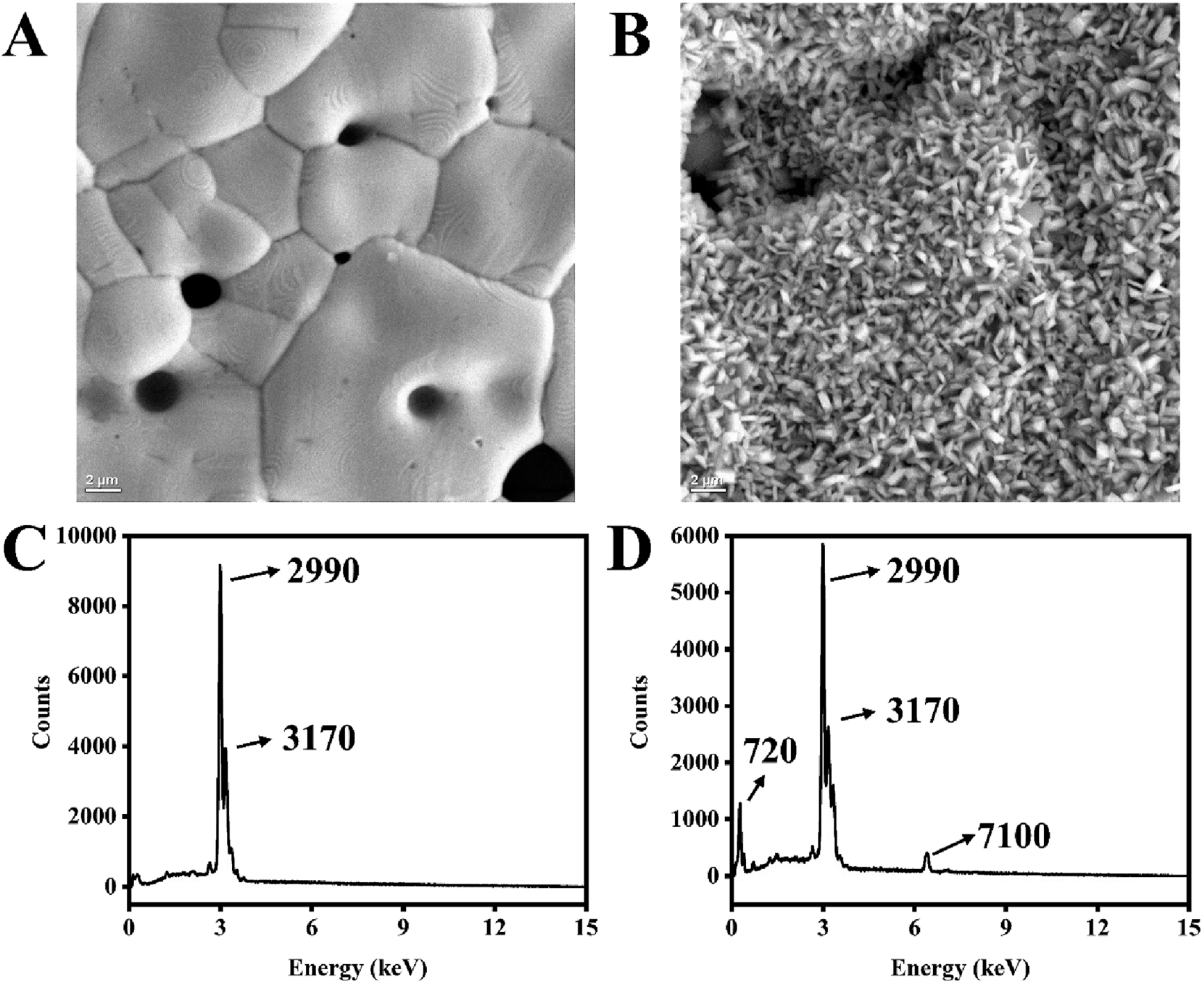
SEM images of the silver electrode surface of BT220 fresh from the box **(A)** and after dipping in a 2 mM ferri/ferrocyanide mixture for 1 h **(B)**. EDX spectrum of the silver electrode surface of BT220 fresh from the box **(C)** and dipping in 2 mM ferri/ferrocyanide mixture for an hour **(D)**.

A mixture of ferricyanide and ferrocyanide is a standard redox probe used during the electrochemical analysis of fabricated biosensors (Chun et al., 2013; Gong et al., 2009; Keighley et al., 2008). The reference electrode used is usually a silver wire encased in a tube filled with KCl solution. However, for BT220, the pseudo-reference silver electrode is exposed to the electrolyte. Ferreira et al. reported that the reference electrode (AT220) is not stable in electrolyte used, therefore researchers implemented an external reference for all their experiments (Ferreira et al., 2021). To verify this, the SPEs were immersed in different solutions, and discoloration was observed on the reference electrode when it was exposed to a 2 mM ferri/ferrocyanide solution for 1 h. The SEM images captured significant changes in the morphology of the reference electrode (Figures 6B–D). The reference electrode exposed to the ferri/ferrocyanide solution showed small, cylinder-like particles densely and evenly distributed on the surface (Figure 6B). Additionally, EDX analysis revealed an iron signal at 7.1 keV (Figure 6D), indicating the presence of Fe compounds in the degraded surface. The carbon peak (at 720 eV) increased significantly after dipping the electrode in the ferri/ferrocyanide mixture, and the normalized signal of silver decreased (from approximately 96%–74%). In contrast, the signals of iron and potassium increased from 0% to 6% and 0%–9%, respectively (Supplementary Tables S1, S2).

#### 3.2.2 An exposure to organic solvent

**FIGURE 7:**
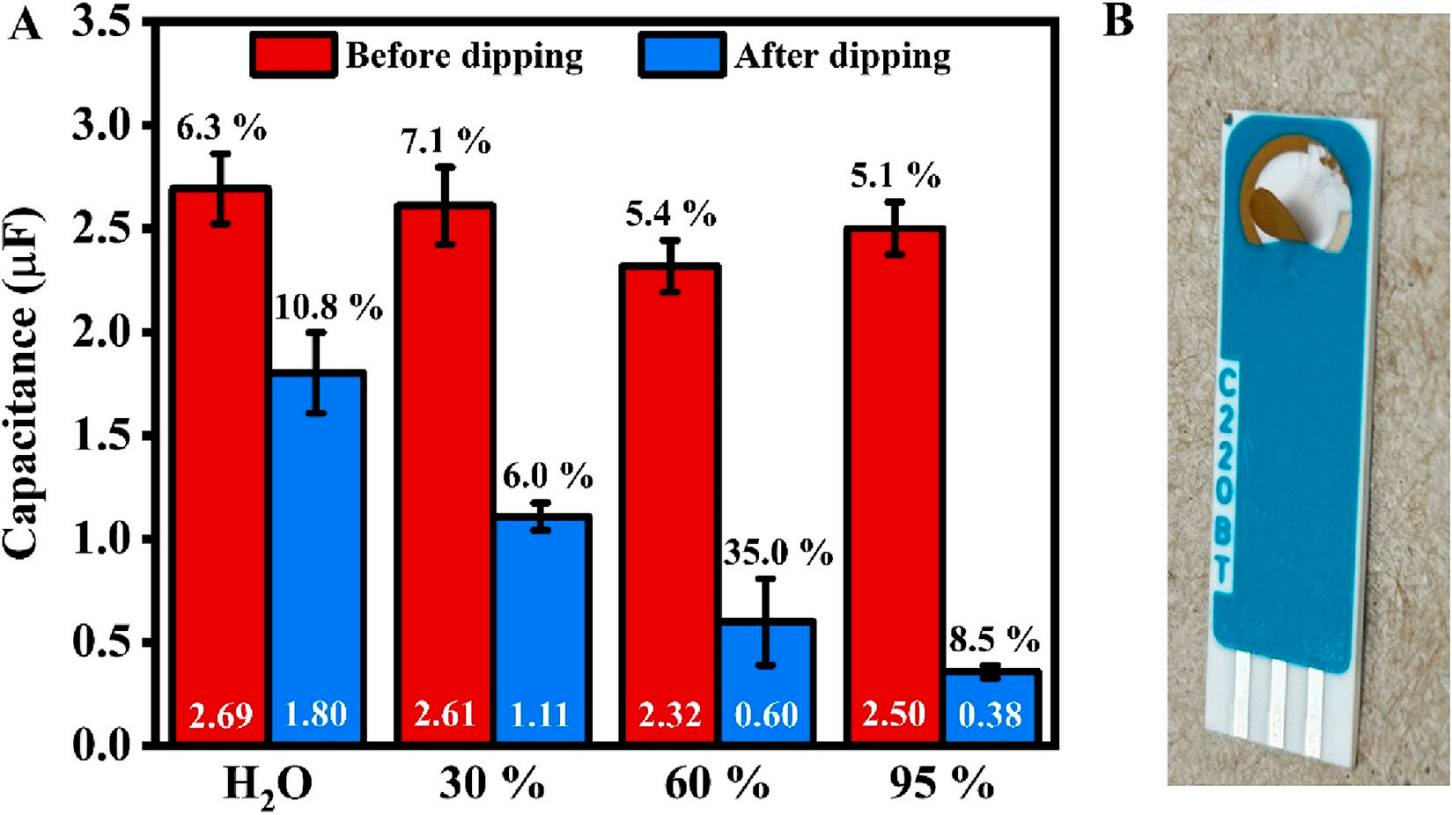
**(A)** Bar chart showing the average values of SPE’s capacitance before (red, left) and after (blue, right) dipping in 30%, 60%, and 95% ethanol solutions for an hour. The values in white at the bottom of the bar represent the average capacitance. In contrast, the percentage values (in black) above indicate the error (n = 5, except for 60% ethanol, with n = 4). **(B)** A photo of the SPE after overnight immersion in a 95% ethanol solution.

Some of the SPE modifications require the use of organic solvents, which are not recommended by the electrode manufacturer. For example, 11-Mercapto-1-undecanol (MCU) is one of the blocking agents that can be potentially used as a gold backfilling solution to prevent the attachment of non-target biomolecules to the gold surface. This is particularly important for SPE-based biosensors intended for use with body fluids that contain additional proteins with a high affinity for gold due to the presence of sulfur-containing moieties (e.g., blood, urine, saliva). For this reason, we also investigated the stability of the electrode in ethanol, a solvent that is compatible with most currently used thiolated blocking agents. A considerable change in capacitance was observed after immersing electrodes in different mixtures of alcohol and water (Figure 7A). The percentage change in capacitance are 33.0%, 57.6%, 74.2%, and 85.7%, respectively, for H_2_O, 30%, 60%, and 95% ethanol solution. Furthermore, after immersion in 95% ethanol for 1 day, the gold layer began to peel off, even with the gentlest rinsing and blow-drying to prepare the SPE for the ECS test (Figure 7B). This indicates that ethanol reacts with the binder used in SPE manufacturing, thereby causing damage. This also explains the considerable decrease in capacitance after dipping the electrodes in alcohol solutions for an hour.

## 4 Conclusion

To optimize the electropolishing process of gold SPE, we have found that (a) with increasing numbers of CV scans, the morphology of most outer deposits becomes very similar, resulting in low RSD and reproducible electrochemically active surface area (b) both the surface area and capacitance increase as the upper potential limit of the CV increases (c) the number of CV cycles during electropolishing should be set to ensure that all electrodes reached the same gold reduction peak current, which provides both the very low RSD for electrochemical quantitate of a baseline electrode (i.e., capacitance and active surface area below 2.9% and 1.9%, respectively).

The reference electrode incorporated in SPE is not stable in ferricyanide/ferrocyanide solutions, which are frequently used as a standard redox probe in electrochemical biosensors. In addition, this type of SPE should not be used in solutions containing ethanol, the solvent commonly used to dissolve thiolate blocking agents (like MCH or MCU). This result has significant implications for researchers using thiolated backfilling agents dissolved in ethanol.

Two practical takeaways from this work that are essential for users of these SPEs (a) the Ag pseudo-reference electrode is unstable in this common redox probe, and (b) these electrodes cannot be exposed to ethanol.

## Data Availability

The datasets presented in this study can be found in online repositories. The names of the repository/repositories and accession number(s) can be found in the article/Supplementary Material.
