# Supplementary material for: Surface analysis of Metrohm BT220 screen-printed electrodes through electrochemical techniques: importance of pretreatment
Source: Front Chem. 2025 Sep 17;13:1602365. doi: 10.3389/fchem.2025.1602365 (PMC12484216; doi:10.3389/fchem.2025.1602365)
Supplement: Supplementary file 1 [file DataSheet2.pdf]

Supplementary Material

1 Supplementary Data

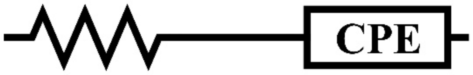

Figure S1: Circuit used to fit all the ECS data. The circuit consists of a resistor and a constant phase element (CPE) connected in series.

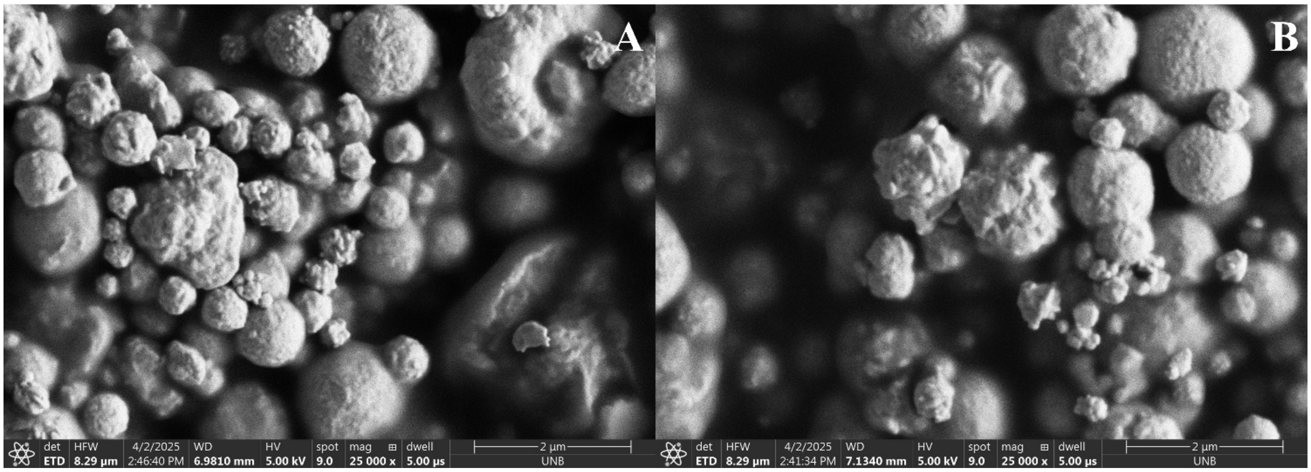

Figure S2: SEM images of gold surface before (A) and after (B) 30 cycles of CV in 0.5 M H_2SO_4 (Potential range: 0.0 V to 1.1 V, scan rate: 0.1 V/s)

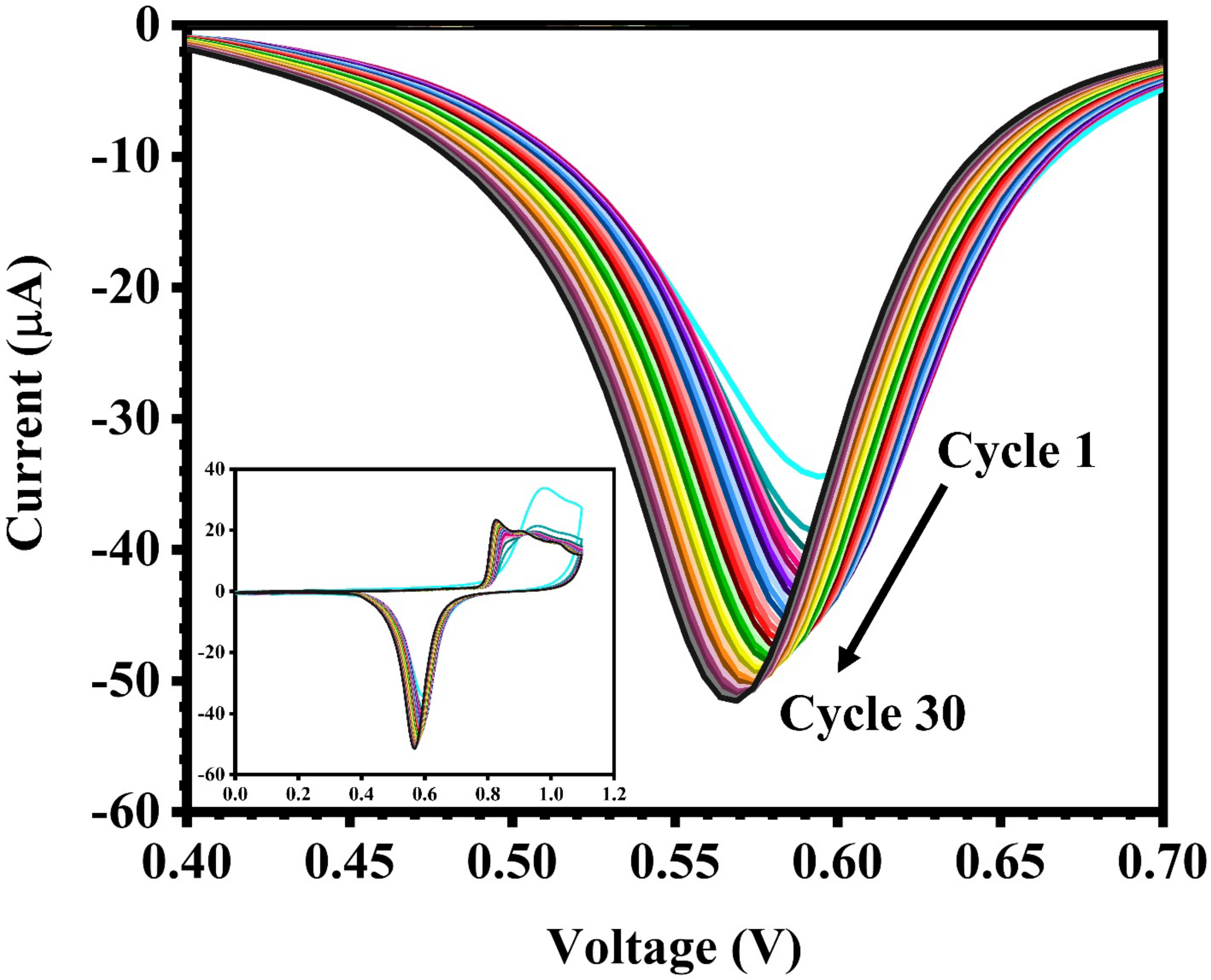

Figure S3: A zoom in image of the reduction peak for an SPE (Electrode 1 from raw data) from cycle 1 (light blue) to cycle 30 (black). The inset shows the full cyclic voltammogram.

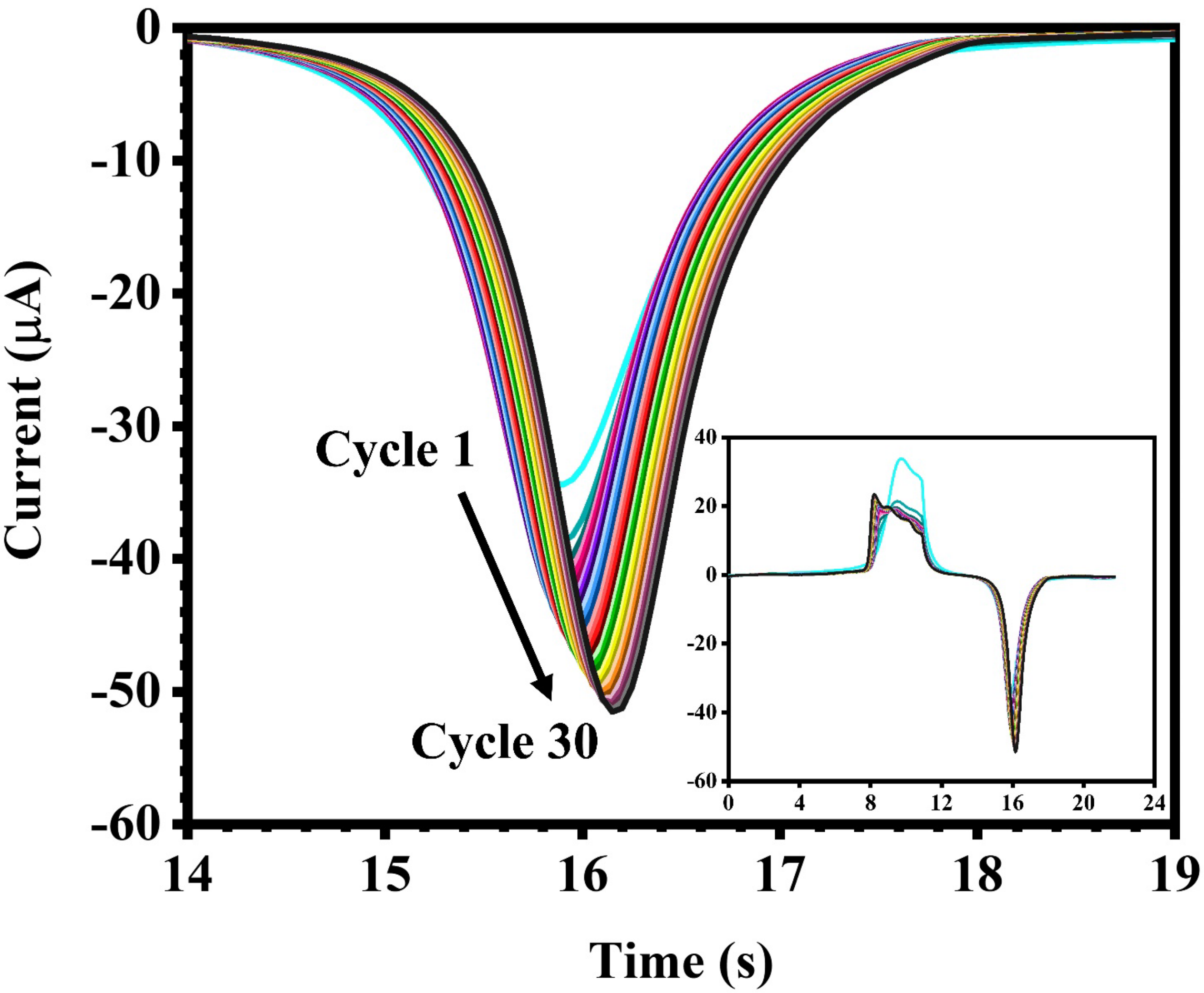

Figure S4: A zoom in image of the reduction peak for an SPE (Electrode 1 from raw data) from cycle 1 (light blue) to cycle 30 (black). This reduction peak was integrated from t = 14 s to t = 18 s for CV with an upper potential of 1.1 V at scan rate of 0.1 V/s. The inset shows the full current vs time graph.

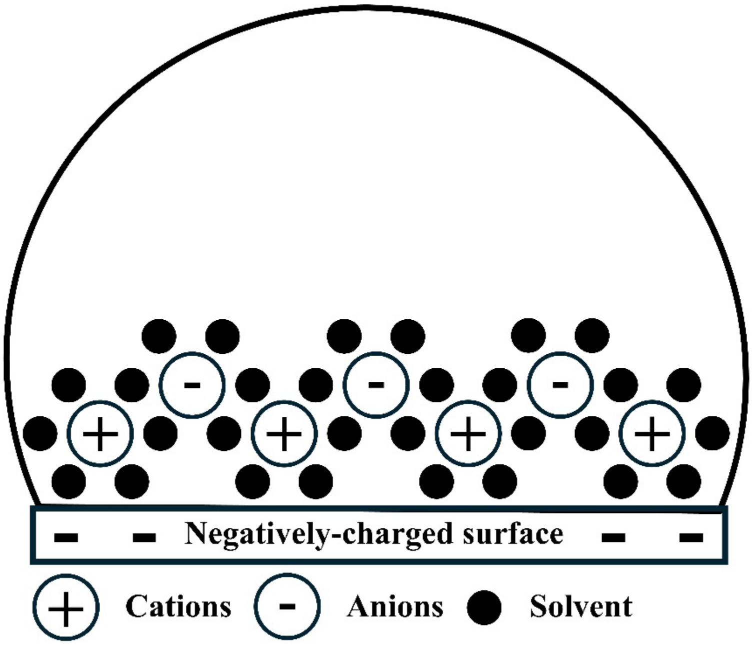

Figure S5: A simplified schematic representation of the formation of double layer on the surface of polarized electrode.

Note (1)**:** The schematic diagram above is oversimplified but it acts as a good starting point for further reading on double layer.

This model was first proposed by Hermann von Helmholtz, where at the surface of a polarized metal-solvent interface, a thin layer of solvated charges is aligned, forming a constant double layer capacitance (Helmholtz layer). In reality, this model is often oversimplified, and much work has been carried out to improve the model. For example, the surface charges do not just reside on the metal-solvent interface. Guoy-Chapman model explained that charge density at the metal-solvent interface is the highest and it dissipates as the distance from the metal surface increases (the diffuse layer). The thickness of the diffuse layer decreases when the potential applied increases, and vice versa. However, the concentration of charged species can become arbitrary large as the distance between the metal-solvent becomes arbitrary small. To compensate for the flaw, Stern model was later introduced by incorporating both Helmholtz model and Guoy-Chapman model. At high potential, almost all charged species are attracted to the surface of the metal (Helmholtz layer); at low potential, the contribution of diffuse layer becomes more prominent.

Table S1: Weight percentage of each element on the silver electrode surface for a BT220 taken out from the box. Five random spots of the silver surface were analyzed to better represent the overall data.

Spots	C	O	Ag	K	Fe	Total
1	1.66	0.00	97.92	0.00	0.42	100
2	2.30	1.67	95.66	0.00	0.37	100
3	2.07	1.94	95.99	0.00	0.00	100
4	2.09	1.02	96.34	0.05	0.49	100
5	2.29	1.12	96.20	0.00	0.39	100

Table S2: Weight percentage of each element on the silver electrode surface for dipping the BT220 in 2 mM ferri/ferrocyanide mixture for an hour. Five random spots of the silver surface were analyzed to better represent the overall data.

Spots	C	O	Ag	K	Fe	Total
1	11.56	0.48	73.27	5.87	8.83	100
2	11.15	0.55	73.90	5.72	8.69	100
3	12.00	0.58	72.91	5.77	8.74	100
4	11.32	0.18	74.35	5.47	8.68	100
5	10.47	0.30	75.62	5.41	8.20	100

References

1. Bard A, Faulkner L, White H. Electrochemical Methods: Fundamentals and Applications, 3rd Edition. 3rd ed. 2022.
